# The role of 53BP1 protein in homology-directed DNA repair: things get a bit complicated

**DOI:** 10.1038/cdd.2016.88

**Published:** 2016-09-30

**Authors:** Michal Malewicz

**Affiliations:** 1 MRC Toxicology Unit, Leicester, UK

Cellular DNA is under a constant threat from metabolism-derived free radicals and exogenous radiation. Therefore, all organisms developed sophisticated DNA repair pathways, which become mobilised in response to DNA damage and serve to promptly restore normal DNA structure.^1^ Double strand DNA breaks (DSBs) pose a particular threat for cellular survival as they impair the progression of important biological processes that occur on DNA such as replication and transcription.^2^ Therefore, cells evolved two mechanisms of DSB repair responsible for efficient DSB removal: non-homologous end joining (NHEJ) and homology-directed repair (HDR). In mammalian cells, NHEJ is mostly active in G1 phase of cell cycle. HDR, on the contrary, is prominent outside of G1 and it is dependent on so-called DNA end resection (generation of single stranded DNA at the free DNA ends), which is a prerequisite for commitment to HDR and which strongly inhibits NHEJ. Mammalian cells perform several types of HDR reactions, which differ in their specific mechanisms, outcomes and specialised protein factors. The most common HDR type is a gene-conversion (GC) reaction, which shows high fidelity, needs a homologous chromosome copy and relies on RAD51 recombinase.^3^ Alternatively, cells could also perform a single-strand annealing type of HDR (SSA), which is highly mutagenic, requires long sequence repeats and depends on RAD52 recombinase. A DSB repair pathway choice is a complex process and mammalian cells evolved an intricate set of its regulators. Prominent among these are 53BP1 adaptor protein and BRCA1 tumor suppressor.^3^ 53BP1 is a large nuclear protein, which is recruited to DSBs via sophisticated cascade of modifications of chromatin surrounding DSBs.^4^ Recently, a structural basis for nucleosome recognition by 53BP1 was uncovered and shown to involve a direct interaction of 53BP1 with specific histone marks and nucleosomal elements.^5^ Accordingly, 53BP1 spreads on chromatin flanking DSBs and is vital in promoting NHEJ pathway by protecting broken DNA ends from extensive resection. The mechanisms by which 53BP1 protein operates have recently become a subject of a very intense research effort.^6^ 53BP1 is not only a critical regulator of mammalian DSB repair but also it has recently emerged as potent antagonist of BRCA1 protein.^7^ BRCA1 in itself is a factor critical to HDR and frequent BRCA1 mutations in breast and ovarian cancers render these cells HDR-deficient. HDR deficiency has been exploited in the clinic as these cells are hypersensitive to inhibition of alternative DNA repair pathways.^8^ For example, BRCA1 mutated cancers are very sensitive to treatment with poly-ADP-ribose polymerase (PARP) inhibitors, as these compounds presumably indirectly trigger generation of DNA lesions, for which HDR repair is essential.^9^ However, resistance to PARP inhibitors frequently occurs in these tumours and could be associated with secondary mutations in 53BP1 pathway resulting in partial HDR restoration. Thus, a thorough understanding of all aspects of 53BP1 action is critically important. Up to this point it has been widely assumed that 53BP1 generally antagonised HDR. A recent report by Ochs *et al.*^10^ challenges that model by proposing a much more refined function for 53BP1 in HDR. The authors established a sophisticated imaging assay suitable for monitoring of DNA damage signalling in large cell populations across various cell cycle stages. Surprisingly, this setting revealed that at least in the late S and G2 phases of the cell cycle 53BP1 promotes GC-HDR and strongly antagonises SSA-HDR. To address the function of 53BP1 in DSB repair without directly depleting its expression, the authors exploited their previous observation that a typical cell can only serve a limited number of DSBs in terms of 53BP1 loading.^11^ Once the DSB number had crossed a certain threshold (typically ca. 5 Gy in their model system; equivalent to ca. 50 DSBs), the DSBs were no longer coated with 53BP1 due to a limited ability of the cell to mark the chromatin with specific ubiquitin conjugates that 53BP1 recognises on chromatin at DSBs. In that case, the DSBs were unable to recruit sufficient 53BP1 and become extensively resected, which in turn promoted SSA mutagenic RAD52-dependent HDR over a conservative GC RAD51-dependent repair. In support of a direct role of 53BP1 in that switch, a specific siRNA-mediated 53BP1 depletion produced a very similar phenotype. Mechanistically, this report^10^ shows reduced RAD51 affinity towards chromatin in the absence of 53BP1 and replacement of RAD51 with RAD52 at DSBs. This is accompanied by a modest reduction in GC-HDR as measured by reporter gene assays and striking up regulation of SSA type of repair. Consequently, at low DSB load, the cells rely on RAD51 for survival, however, with increasing DSB load the cells become progressively dependent on RAD52. Thus, 53BP1 role in HDR is to facilitate limited resection suitable for GC-HDR and suppress extensive resection compatible with SSA (Figure 1). There are several important implications of this work. Notably. 53BP1 loading on chromatin depends on the overall activity of various specialised ubiquitin ligases (e.g. RNF168) and their steady state levels, which could be controlled by various other signalling pathways.^11^ It thus appears possible that 53BP1-dependent DSB regulation could have various thresholds depending on cell type and genetic context. As the authors themselves noted, it might potentially explain previously observed lack of the influence of 53BP1 loss on GC-HDR in some settings.^10^ It is nevertheless worth noticing that the cellular DNA repair regulation may have a certain cell type specific dimension and it warrants careful interpretation and comparison of the data obtained in various cellular systems. Another important implication of this work relates to biology of BRCA1-deficient cancers. Such tumours could potentially become even more aggressive once secondary mutations in 53BP1 pathway arose with subsequent restoration of the error-prone RAD52-dependent HDR.^12^ However, this gloomy outlook could be counterbalanced by a therapeutic possibility the RAD52 dependency creates. Efforts need to be directed to exploit RAD52 as a potential drug target in a clinical setting.

There are several additional questions raised by the findings of Ochs*et al*. For example it is not entirely clear what circumstances could lead to 53BP1 pathway saturation under physiological conditions where only a handful of endogenous DSBs typically arise. Could this phenomenon apply to situations such as cancer radiotherapy where larger amounts of DSBs are routinely induced in the cancerous tissue? Furthermore, would there be any benefit for the cell to switch to SSA in the face of very high DSB numbers? A general priority for a cell is to re-establish the linear unperturbed DNA structure, the fidelity of repair being of a secondary importance. This is because the presence of DNA breaks threatens cellular survival, whereas the presence of mutations in mammalian genome can frequently be tolerated due to poor gene density. A cell might therefore choose to switch to SSA should that process achieve a faster or more complete DNA repair. It would be also very interesting to investigate the known 53BP1 effector proteins for their role in RAD52 activity suppression. For example, proteins such as Rif1,^13^ PTIP^14^ and Rev7/MAD2L2^15, 16^ all have all been implicated in execution of 53BP1 functions of controlling DNA end resection. On the other hand, RAD51 filament formation *in vivo* is antagonised at various regulatory levels by several distinct classes of proteins. These are factors such as Poltheta^2^ (an error-prone polymerase involved in mutagenic NHEJ) and various antirecombinases such as RECQL5 and BLM.^2^ It is conceivable that 53BP1 would intersect with some of these critical HDR regulators to execute its function in this DNA repair pathway.

## Figures and Tables

**Figure 1:**
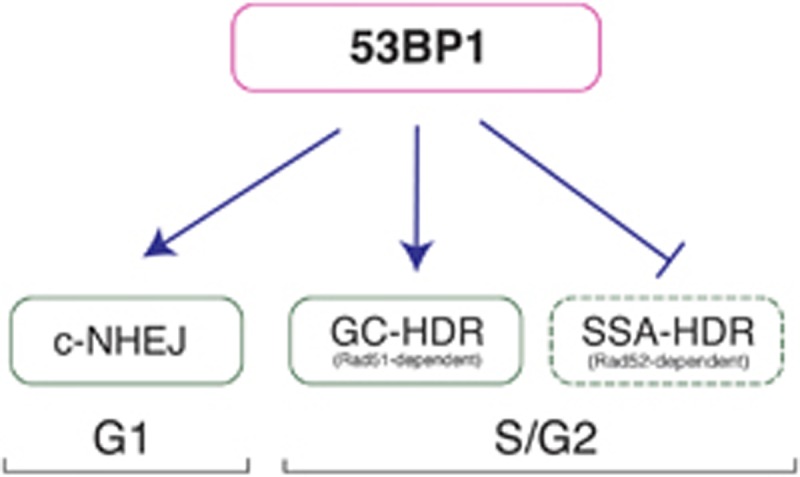
Complex role for 53BP1 protein in regulation of mammalian DNA double strand break repair pathway choice. 53BP1 promotes classical non-homologous end joining (c-NHEJ) DNA repair pathway in G1 phase of cell cycle. In S- and G2-phases of the cell cycle, 53BP1 promotes high fidelity RAD51-dependent gene conversion homology-directed DNA repair (GC-HDR) and strongly antagonises the mutagenic RAD52-dependent single strand annealing type of homology-directed DNA repair (SSA-HDR)
